# 2-(Methoxy­carbon­yl)anilinium dihydrogen phosphate

**DOI:** 10.1107/S1600536809025252

**Published:** 2009-07-04

**Authors:** Muhammad Shafiq, Islam Ullah Khan, Muhammad Nadeem Arshad, Muneeb Hayat Khan, Jim Simpson

**Affiliations:** a Materials Chemistry laboratory, Department of Chemistry, GC University, Lahore, Pakistan; b Department of Chemistry, University of Otago, PO Box 56, Dunedin, New Zealand

## Abstract

The title compound, C_8_H_10_NO_2_^+^·H_2_PO_4_^−^, is a derivative of the naturally occurring compound methyl­anthranilate. The asymmetric unit comprises the 2-(methoxy­carbon­yl)anilinium cation and the dihydrogen phosphate anion. In the cation, the dihedral angle between the benzene ring plane and that through the methyl ester substituent is 22.94 (9)°. In the crystal, adjacent cations and anions form dimers through N—H⋯O and O—H⋯O hydrogen bonds, respectively. Additional N—H⋯O and C—H⋯O contacts result in a network of cation and anion dimers stacked down the *b* axis.

## Related literature

For thia­zine-related heterocycles see: Shafiq *et al.* (2009*a* ▶). For related structures, see: Gel’mbol’dt *et al.* (2006 ▶); Ma *et al.* (2005 ▶); Shafiq *et al.* (2008 ▶, 2009*b* ▶).
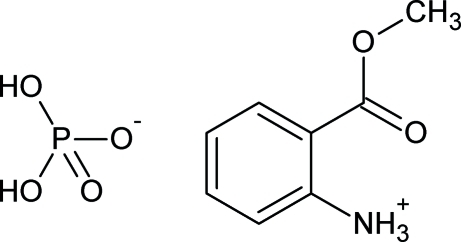


## Experimental

### 

#### Crystal data

C_8_H_10_NO_2_^+^·H_2_PO_4_^−^
*M* *_r_* = 249.16
Monoclinic, 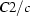

*a* = 20.939 (3) Å
*b* = 4.7880 (5) Å
*c* = 22.283 (4) Å
β = 114.970 (5)°
*V* = 2025.2 (5) Å^3^
*Z* = 8
Mo *K*α radiation
μ = 0.29 mm^−1^
*T* = 296 K
0.39 × 0.21 × 0.17 mm

#### Data collection

Bruker Kappa APEXII CCD diffractometer
Absorption correction: multi-scan (*SADABS*; Bruker, 2005 ▶) *T*_min_ = 0.928, *T*_max_ = 0.958
10812 measured reflections
2413 independent reflections
2078 reflections with *I* > 2σ(*I*)
*R*_int_ = 0.035

#### Refinement

*R*[*F* ^2^ > 2σ(*F* ^2^)] = 0.037
*wR*(*F* ^2^) = 0.102
*S* = 1.07
2413 reflections
153 parameters
H atoms treated by a mixture of independent and constrained refinement
Δρ_max_ = 0.29 e Å^−3^
Δρ_min_ = −0.48 e Å^−3^

### 

Data collection: *APEX2* (Bruker, 2007 ▶); cell refinement: *SAINT* (Bruker, 2007 ▶); data reduction: *SAINT*; program(s) used to solve structure: *SHELXS97* (Sheldrick, 2008 ▶); program(s) used to refine structure: *SHELXL97* (Sheldrick, 2008 ▶); molecular graphics: *SHELXTL* (Sheldrick, 2008 ▶) and *Mercury* (Macrae *et al.*, 2006 ▶); software used to prepare material for publication: *SHELXL97*, *enCIFer* (Allen *et al.*, 2004 ▶), *PLATON* (Spek, 2009 ▶) and *publCIF* (Westrip, 2009 ▶).

## Supplementary Material

Crystal structure: contains datablocks global, I. DOI: 10.1107/S1600536809025252/bt2987sup1.cif

Structure factors: contains datablocks I. DOI: 10.1107/S1600536809025252/bt2987Isup2.hkl

Additional supplementary materials:  crystallographic information; 3D view; checkCIF report

## Figures and Tables

**Table 1:** Hydrogen-bond geometry (Å, °)

*D*—H⋯*A*	*D*—H	H⋯*A*	*D*⋯*A*	*D*—H⋯*A*
C4—H4⋯O5	0.93	2.68	3.387 (2)	133
C4—H4⋯O6	0.93	2.71	3.590 (2)	158
C3—H3⋯O2^i^	0.93	2.72	3.609 (2)	162
N1—H1*A*⋯O3^ii^	0.89	1.93	2.8218 (18)	178
N1—H1*A*⋯O5^ii^	0.89	2.68	3.190 (2)	118
N1—H1*C*⋯O3^iii^	0.89	2.01	2.9005 (18)	175
N1—H1*B*⋯O3^iv^	0.89	2.09	2.9400 (17)	160
O5—H5*O*⋯O6^v^	0.78 (2)	1.83 (2)	2.6021 (17)	170 (2)
O4—H4*O*⋯O6^vi^	0.72 (2)	1.88 (2)	2.6015 (17)	179 (2)

Symmetry codes: (i) 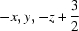; (ii) 
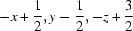
; (iii) 
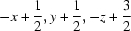
; (iv) 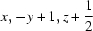; (v) 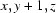; (vi) 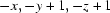.

**Fig. 1.**
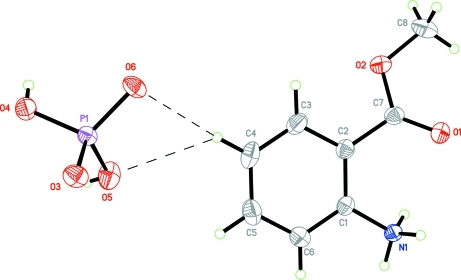
The asymmetric unit of (I) with displacement ellipsoids for the non-hydrogen atoms drawn at the 50% probability level. Hydrogen bonds are drawn as dashed lines.

**Fig. 2.**
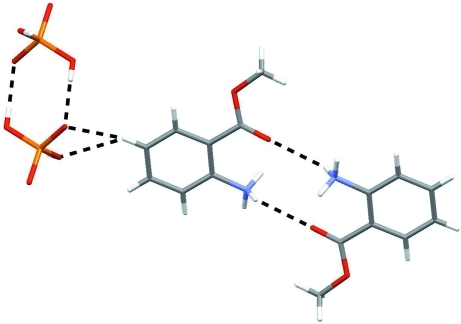
Dimers formed by the cations and anions in the structure of (I) with hydrogen bonds drawn as dashed lines.

**Fig. 3.**
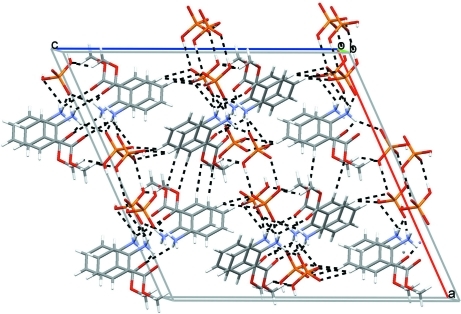
Crystal packing of (I) viewed down the b axis

## supplementary crystallographic information

### Comment

Methylanthranilite is a naturally occurring compound which has been used in food flavoring, as a fragrance additive and a bird repellent. Our group has been involved in the synthesis of heterocyclic molecules related to benzothiazine (Shafiq *et al.*, 2009*a*) in which the title compound has been used as a starting material (Shafiq *et al.*, 2008; Shafiq *et al.*, 2009*b*). Herein we report the structure of title compound which was obtained during the synthesis of methylanthranilate Schiff base derivatives.

The asymmetric unit comprises the 2-(methoxycarbonyl)anilinium cation and the dihydrogen phosphate anion linked by bifurcated C4–H4···O5 and C4—H4···O6 interactions, Fig. 1. In the cation the dihedral angle between the C1···C6 benzene ring plane and that through the C2,C7(O1),O2,C8 atoms of the methyl ester substituent is 22.94 (9) °. Bond distances within the molecule are normal  and similar to those observed in comparable structures (Gel'mbol'dt *et al.*, 2006; Ma *et al.*, 2005). In the crystal, adjacent cations and anions form dimers through N1—H1A···O1 and O4—H4O···O6 hydrogen bonds respectively, Fig. 2. Additional N—H···O and C—H···O contacts result in a network of cation and anion dimers stacked down the *b* axis, Fig. 3.

### Experimental

The title compound was obtained during the synthesis of Schiff base of methyl anthranilate. Methyl anthranilite (0.5 g, 0.0 mol) was dissolved in methanol (5 ml). A few drops of polyphosphoric acid were added to the above solution to precipitate the product   which was recrystalized from methanol.

### Refinement

The H atoms bound to N1, O4 and O5 were located in a difference Fourier map and their coordinates were refined with *U*_iso_= 1.5*U*_eq_ (N) and 1.2*U*_eq_ (O). All other H-atoms were placed in calculated positions and refined using a riding model with d(C—H) = 0.93 Å, *U*_iso_ = 1.2*U*_eq_ (C) for aromatic and 0.96 Å, *U*_iso_ = 1.5*U*_eq_ (C) for CH_3_ H atoms.

### Crystal data

**Table:**

C_8_H_10_NO_2_^+^·H_2_PO_4_^−^	*F*(000) = 1040
*M**_r_* = 249.16	*D*_x_ = 1.634 Mg m^−^^3^
Monoclinic, *C*2/*c*	Mo *K*α radiation, λ = 0.71073 Å
Hall symbol: -C 2yc	Cell parameters from 5124 reflections
*a* = 20.939 (3) Å	θ = 2.2–27.9°
*b* = 4.7880 (5) Å	µ = 0.29 mm^−^^1^
*c* = 22.283 (4) Å	*T* = 296 K
β = 114.970 (5)°	Needle, colourless
*V* = 2025.2 (5) Å^3^	0.39 × 0.21 × 0.17 mm
*Z* = 8	

### Data collection

**Table:**

Bruker Kappa APEXII CCD diffractometer	2413 independent reflections
Radiation source: fine-focus sealed tube	2078 reflections with *I* > 2σ(*I*)
graphite	*R*_int_ = 0.035
φ and ω scans	θ_max_ = 27.9°, θ_min_ = 3.5°
Absorption correction: multi-scan (*SADABS*; Bruker, 2005)	*h* = −27→27
*T*_min_ = 0.928, *T*_max_ = 0.958	*k* = −4→6
10812 measured reflections	*l* = −29→27

### Refinement

**Table:**

Refinement on *F*^2^	Primary atom site location: structure-invariant direct methods
Least-squares matrix: full	Secondary atom site location: difference Fourier map
*R*[*F*^2^ > 2σ(*F*^2^)] = 0.037	Hydrogen site location: inferred from neighbouring sites
*wR*(*F*^2^) = 0.102	H atoms treated by a mixture of independent and constrained refinement
*S* = 1.07	*w* = 1/[σ^2^(*F*_o_^2^) + (0.0575*P*)^2^ + 1.6713*P*] where *P* = (*F*_o_^2^ + 2*F*_c_^2^)/3
2413 reflections	(Δ/σ)_max_ = 0.001
153 parameters	Δρ_max_ = 0.29 e Å^−^^3^
0 restraints	Δρ_min_ = −0.48 e Å^−^^3^

### Special details

**Table:**

Geometry. All e.s.d.'s (except the e.s.d. in the dihedral angle between two l.s. planes) are estimated using the full covariance matrix. The cell e.s.d.'s are taken into account individually in the estimation of e.s.d.'s in distances, angles and torsion angles; correlations between e.s.d.'s in cell parameters are only used when they are defined by crystal symmetry. An approximate (isotropic) treatment of cell e.s.d.'s is used for estimating e.s.d.'s involving l.s. planes.
Refinement. Refinement of *F*^2^ against ALL reflections. The weighted *R*-factor *wR* and goodness of fit *S* are based on *F*^2^, conventional *R*-factors *R* are based on *F*, with *F* set to zero for negative *F*^2^. The threshold expression of *F*^2^ > σ(*F*^2^) is used only for calculating *R*-factors(gt) *etc*. and is not relevant to the choice of reflections for refinement. *R*-factors based on *F*^2^ are statistically about twice as large as those based on *F*, and *R*- factors based on ALL data will be even larger.

### Fractional atomic coordinates and isotropic or equivalent isotropic displacement parameters (Å^2^)

**Table:**

	*x*	*y*	*z*	*U*_iso_*/*U*_eq_	
N1	0.24287 (7)	0.4968 (3)	0.93401 (6)	0.0200 (3)	
H1A	0.2695	0.3443	0.9473	0.030*	
H1B	0.2127	0.5014	0.9526	0.030*	
H1C	0.2702	0.6478	0.9460	0.030*	
C1	0.20379 (8)	0.4920 (3)	0.86203 (7)	0.0202 (3)	
C2	0.14685 (8)	0.3118 (3)	0.83134 (7)	0.0227 (3)	
C3	0.11163 (9)	0.3157 (4)	0.76239 (8)	0.0300 (4)	
H3	0.0730	0.1996	0.7412	0.036*	
C4	0.13337 (10)	0.4893 (4)	0.72527 (9)	0.0326 (4)	
H4	0.1102	0.4864	0.6793	0.039*	
C5	0.18948 (10)	0.6671 (4)	0.75648 (9)	0.0322 (4)	
H5	0.2038	0.7857	0.7315	0.039*	
C6	0.22462 (9)	0.6700 (4)	0.82496 (8)	0.0273 (4)	
H6	0.2622	0.7916	0.8459	0.033*	
C7	0.12565 (8)	0.1075 (3)	0.86989 (8)	0.0225 (3)	
C8	0.03453 (11)	−0.1887 (5)	0.86660 (11)	0.0433 (5)	
H8A	0.0609	−0.1795	0.9138	0.065*	
H8B	0.0402	−0.3700	0.8511	0.065*	
H8C	−0.0144	−0.1566	0.8555	0.065*	
O1	0.16391 (6)	0.0261 (3)	0.92428 (6)	0.0273 (3)	
O2	0.05989 (7)	0.0218 (3)	0.83567 (7)	0.0380 (3)	
P1	0.10739 (2)	0.56312 (8)	0.531833 (19)	0.01826 (13)	
O3	0.17510 (6)	0.5066 (2)	0.52576 (6)	0.0248 (3)	
O4	0.05239 (6)	0.6781 (3)	0.46425 (6)	0.0262 (3)	
H4O	0.0163 (12)	0.681 (5)	0.4598 (10)	0.031*	
O5	0.12078 (7)	0.8011 (3)	0.58427 (6)	0.0323 (3)	
H5O	0.1063 (13)	0.948 (5)	0.5709 (11)	0.039*	
O6	0.07879 (6)	0.3146 (2)	0.55400 (6)	0.0255 (3)	

### Atomic displacement parameters (Å^2^)

**Table:**

	*U*^11^	*U*^22^	*U*^33^	*U*^12^	*U*^13^	*U*^23^
N1	0.0199 (6)	0.0202 (6)	0.0191 (6)	−0.0008 (5)	0.0075 (5)	−0.0015 (5)
C1	0.0202 (7)	0.0201 (7)	0.0196 (7)	0.0040 (6)	0.0076 (6)	−0.0003 (6)
C2	0.0230 (8)	0.0219 (7)	0.0215 (7)	0.0022 (6)	0.0079 (6)	0.0005 (6)
C3	0.0277 (9)	0.0334 (9)	0.0222 (8)	−0.0011 (7)	0.0038 (6)	−0.0010 (7)
C4	0.0353 (10)	0.0407 (10)	0.0194 (8)	0.0074 (8)	0.0091 (7)	0.0046 (7)
C5	0.0365 (10)	0.0354 (9)	0.0294 (9)	0.0048 (8)	0.0185 (7)	0.0085 (8)
C6	0.0277 (8)	0.0260 (8)	0.0295 (8)	−0.0016 (7)	0.0134 (7)	0.0009 (7)
C7	0.0219 (7)	0.0202 (7)	0.0235 (8)	−0.0008 (6)	0.0077 (6)	−0.0031 (6)
C8	0.0388 (11)	0.0397 (11)	0.0495 (12)	−0.0184 (9)	0.0167 (9)	0.0013 (9)
O1	0.0257 (6)	0.0293 (6)	0.0241 (6)	0.0007 (5)	0.0078 (5)	0.0036 (5)
O2	0.0268 (7)	0.0436 (8)	0.0329 (7)	−0.0137 (6)	0.0022 (5)	0.0073 (6)
P1	0.0173 (2)	0.0154 (2)	0.0226 (2)	0.00185 (14)	0.00890 (16)	0.00206 (14)
O3	0.0181 (6)	0.0250 (6)	0.0324 (6)	0.0032 (4)	0.0118 (5)	0.0025 (5)
O4	0.0179 (5)	0.0338 (7)	0.0261 (6)	0.0029 (5)	0.0086 (5)	0.0069 (5)
O5	0.0466 (8)	0.0189 (6)	0.0277 (6)	0.0072 (5)	0.0122 (6)	0.0001 (5)
O6	0.0234 (6)	0.0186 (5)	0.0363 (6)	0.0010 (4)	0.0142 (5)	0.0060 (5)

### Geometric parameters (Å, °)

**Table:**

N1—C1	1.4609 (19)	C6—H6	0.9300
N1—H1A	0.8900	C7—O1	1.2010 (19)
N1—H1B	0.8900	C7—O2	1.326 (2)
N1—H1C	0.8900	C8—O2	1.443 (2)
C1—C6	1.379 (2)	C8—H8A	0.9600
C1—C2	1.395 (2)	C8—H8B	0.9600
C2—C3	1.396 (2)	C8—H8C	0.9600
C2—C7	1.488 (2)	P1—O3	1.5045 (12)
C3—C4	1.379 (3)	P1—O6	1.5062 (12)
C3—H3	0.9300	P1—O4	1.5597 (12)
C4—C5	1.378 (3)	P1—O5	1.5702 (13)
C4—H4	0.9300	O4—H4O	0.72 (2)
C5—C6	1.386 (2)	O5—H5O	0.78 (2)
C5—H5	0.9300		
			
C1—N1—H1A	109.5	C1—C6—C5	119.85 (16)
C1—N1—H1B	109.5	C1—C6—H6	120.1
H1A—N1—H1B	109.5	C5—C6—H6	120.1
C1—N1—H1C	109.5	O1—C7—O2	124.62 (16)
H1A—N1—H1C	109.5	O1—C7—C2	124.15 (15)
H1B—N1—H1C	109.5	O2—C7—C2	111.21 (14)
C6—C1—C2	120.64 (15)	O2—C8—H8A	109.5
C6—C1—N1	118.39 (14)	O2—C8—H8B	109.5
C2—C1—N1	120.97 (14)	H8A—C8—H8B	109.5
C1—C2—C3	118.45 (15)	O2—C8—H8C	109.5
C1—C2—C7	121.69 (14)	H8A—C8—H8C	109.5
C3—C2—C7	119.77 (15)	H8B—C8—H8C	109.5
C4—C3—C2	120.91 (17)	C7—O2—C8	116.33 (15)
C4—C3—H3	119.5	O3—P1—O6	114.06 (7)
C2—C3—H3	119.5	O3—P1—O4	108.44 (7)
C5—C4—C3	119.79 (16)	O6—P1—O4	111.21 (7)
C5—C4—H4	120.1	O3—P1—O5	108.56 (7)
C3—C4—H4	120.1	O6—P1—O5	107.47 (7)
C4—C5—C6	120.33 (17)	O4—P1—O5	106.83 (7)
C4—C5—H5	119.8	P1—O4—H4O	116.4 (17)
C6—C5—H5	119.8	P1—O5—H5O	117.1 (18)
			
C6—C1—C2—C3	0.1 (2)	N1—C1—C6—C5	178.83 (15)
N1—C1—C2—C3	−179.80 (15)	C4—C5—C6—C1	0.7 (3)
C6—C1—C2—C7	176.75 (15)	C1—C2—C7—O1	−21.1 (2)
N1—C1—C2—C7	−3.1 (2)	C3—C2—C7—O1	155.53 (17)
C1—C2—C3—C4	1.3 (3)	C1—C2—C7—O2	160.61 (16)
C7—C2—C3—C4	−175.43 (16)	C3—C2—C7—O2	−22.8 (2)
C2—C3—C4—C5	−1.7 (3)	O1—C7—O2—C8	−1.5 (3)
C3—C4—C5—C6	0.7 (3)	C2—C7—O2—C8	176.80 (16)
C2—C1—C6—C5	−1.1 (3)		

### Hydrogen-bond geometry (Å, °)

**Table:**

*D*—H···*A*	*D*—H	H···*A*	*D*···*A*	*D*—H···*A*
C4—H4···O5	0.93	2.68	3.387 (2)	133
C4—H4···O6	0.93	2.71	3.590 (2)	158
C3—H3···O2^i^	0.93	2.72	3.609 (2)	162
N1—H1A···O3^ii^	0.89	1.93	2.8218 (18)	178
N1—H1A···O5^ii^	0.89	2.68	3.190 (2)	118
N1—H1C···O3^iii^	0.89	2.01	2.9005 (18)	175
N1—H1B···O3^iv^	0.89	2.09	2.9400 (17)	160
O5—H5O···O6^v^	0.78 (2)	1.83 (2)	2.6021 (17)	170 (2)
O4—H4O···O6^vi^	0.72 (2)	1.88 (2)	2.6015 (17)	179 (2)

Symmetry codes: (i) −*x*, *y*, −*z*+3/2; (ii) −*x*+1/2, *y*−1/2, −*z*+3/2; (iii) −*x*+1/2, *y*+1/2, −*z*+3/2; (iv) *x*, −*y*+1, *z*+1/2; (v) *x*, *y*+1, *z*; (vi) −*x*, −*y*+1, −*z*+1.
